# The Coronavirus PEDV Evades Type III Interferon Response Through the miR-30c-5p/SOCS1 Axis

**DOI:** 10.3389/fmicb.2020.01180

**Published:** 2020-05-22

**Authors:** Changlin Wang, Lingling Shan, Shuxin Qu, Mei Xue, Keliang Wang, Fang Fu, Lu Wang, Ziqi Wang, Li Feng, Wanhai Xu, Pinghuang Liu

**Affiliations:** ^1^Department of Urology, the Fourth Affiliated Hospital of Harbin Medical University, Harbin, China; ^2^State Key Laboratory of Veterinary Biotechnology, Harbin Veterinary Research Institute, Chinese Academy of Agricultural Sciences, Harbin, China

**Keywords:** coronavirus, PEDV, IFN-λ, SOCS1, microRNA, miR-30c-5p

## Abstract

Porcine epidemic diarrhea virus (PEDV) is an economically important pathogen that has evolved several mechanisms to evade type I IFN responses. Type III interferon (IFN-λ), an innate cytokine that primarily targets the mucosal epithelia, is critical in fighting mucosal infection in the host and has been reported to potently inhibit PEDV infection *in vitro*. However, how PEDV escapes IFN-λ antiviral response remains unclear. In this study, we found that PEDV infection induced significant IFN-λ expression in type I IFN-defective Vero E6 cells, but virus-induced endogenous IFN-λ did not reduce PEDV titers. Moreover, we demonstrated that PEDV escaped IFN-λ responses by substantially upregulating the suppressor of cytokine signaling protein 1 (SOCS1) expression, which impaired the induction of IFN-stimulated genes (ISGs) and dampened the IFN-λ antiviral response and facilitated PEDV replication in Vero E6 cells. We further showed that PEDV infection increased SOCS1 expression by decreasing host miR-30c-5p expression. MiR-30c-5p suppressed SOCS1 expression through targeting the 3′ untranslated region (UTR) of SOCS1. The inhibition of IFN-λ elicited ISGs expression by SOCS1 was specifically rescued by overexpression of miR-30c-5p. Collectively, our findings identify a new strategy by PEDV to escape IFN-λ-mediated antiviral immune responses by engaging the SOCS1/miR-30c axis, thus improving our understanding of its pathogenesis.

## Introduction

Porcine epidemic diarrhea virus (PEDV), a member of the *Alphacoronavirus* family, is an enteropathogenic coronavirus with economic importance (Madson et al., 2014; Wang et al., 2014; Zhang and Yoo, 2016). PEDV infection in newborn piglets is characterized by vomiting, anorexia, watery diarrhea, and dehydration (Song and Park, 2012). The virus primarily infects small intestinal epithelial cells *in vivo* and causes high morbidity and mortality in piglets (Li et al., 2012). Interferons (IFNs) are the key components of innate immunity in response to viral infection (Zhang et al., 2018). Among three types of IFNs (types I, II, and III), type III IFN-lambda (IFN-λ) primarily acts on mucosal surfaces, including epithelial surfaces of the liver, respiratory, and gastrointestinal systems, and plays vital roles in controlling viral infection within mucosal surfaces (Mordstein et al., 2010; Pott et al., 2011; Lazear et al., 2015). We and other groups previously demonstrated that porcine IFN-λdisplays powerful antiviral activity against PEDV infection in both Vero E6 cells and porcine intestinal epithelia (Li et al., 2017, 2019). PEDV has evolved multiple strategies to escape IFN responses, including the degradation of STAT1 and the suppression of type I IFN production (Guo et al., 2016). Although type I and type III IFNs have a large overlap in the spectrum of induced antiviral ISG responses, recent studies demonstrated that type III IFN is a critical non-redundant antiviral mediator of type I IFNs in the GI tract and elicits a unique transcriptional profile that does not completely overlap with that induced by IFN-α (Wells and Coyne, 2018). It is necessary to clarify how PEDV evades type III IFN following infection.

Unlike ample studies reporting that PEDV escapes type I IFNs, limited studies demonstrate that PEDV escapes IFN-λ response. PEDV suppresses IRF1-mediated type III IFN responses by reducing the number of peroxisomes and counteracting type III IFN response by PEDV nsp15 endoribonuclease (Zhang et al., 2018; Deng et al., 2019). Deng et al. showed that type I and type III IFNs exhibit different modulation in response to PEDV infection and that the discrepancy of type I and type III IFN responses is independent of PEDV endoribonuclease activity (Deng et al., 2019), suggesting that there are distinct strategies to modify host type I and type III IFN responses during PEDV infection. Because cells generally produce both type I and type III IFNs in response to viral infection, it is challenging to elucidate how viruses escape IFN-λ response separately to type I response. In this study, we used Vero cells, a cell line with a defective function, to produce endogenous type I IFNs. Vero cells are widely used as an *in vitro* model to study the interactions between viruses and hosts including PEDV. We and others reported that Vero cells respond well to both porcine type I and type III IFNs (Guo et al., 2016; Shen et al., 2016; Li et al., 2017). IFN-λ is rapidly produced after infection and following engagement with its receptor induces IFN-stimulated gene (ISG) expression to mediate antiviral activity (Kotenko et al., 2003; Dellgren et al., 2009; Lazear et al., 2015). Binding of IFN-λ to its receptor, which consists of two subunits, IFN-λR1 and IL-10R2, leads to activation of JAK1 and Tyk2, which mediates the phosphorylation of STAT1 and STAT2 proteins (Sheppard et al., 2003; Palma-Ocampo et al., 2015). The suppressor of cytokine signaling protein 1 (SOCS1), a negative regulator of Janus family kinase (JAK) signal transducer, simultaneously binds the receptors and JAKs and prevents STATs from accessing the receptor kinase complex (de Weerd and Nguyen, 2012; Palma-Ocampo et al., 2015). Previous reports demonstrated that SOCS1 is an inducible negative regulator of IFN-λ-induced gene expression *in vivo* (Blumer et al., 2017). SOCS1 was also associated with DENV-2 escape from IFN-λ response during infection (Palma-Ocampo et al., 2015). However, the role of SOCS1 during PEDV infection remains unclear.

MicroRNAs (miRNAs), as important post-transcriptional modulators of gene expression, participate in modulating the host innate and adaptive immune responses in response to pathogen invasion (Baltimore et al., 2008; Gottwein and Cullen, 2008; O'Neill et al., 2011). Increasing evidence has shown that miRNAs of viral and cellular origin can help viruses evade host immune responses by targeting critical components in the host immune system (Cullen, 2006; Sullivan et al., 2006; Kincaid and Sullivan, 2012). For example, miR-30c is a potent negative regulator of type I IFN signaling by targeting JAK1, resulting in the enhancement of PRRSV infection (Zhang et al., 2016). The miR-30 family is a well-studied host miRNA that plays an important role in viral infection by modulating IFN signaling (Zhu et al., 2014; Zhang et al., 2016; Liu et al., 2018; Ma et al., 2018). Our previous study revealed that TGEV escapes type I IFN response by engaging the IRE1-miR-30a-5p/SOCS1/3 axis (Ma et al., 2018). The potential role of miRNAs in coronavirus escape from IFN-λ response remains elusive.

In this study, we showed that PEDV escaped IFN-λ responses by upregulating SOCS1 expression in type I IFN-defective Vero E6 cells. In addition, we demonstrated that PEDV infection increased SOCS1 expression by decreasing the expression of host miR-30c-5p, which modulates SOCS1 expression by specifically targeting the 3′ UTR of SOCS1. Our findings identify a new strategy by PEDV to escape IFN-λ-mediated host innate immune defenses.

## Materials and Methods

### Cells and Viruses

African green monkey kidney cells (Vero E6 cells) were stocked by our laboratory and grown in DMEM (Gibco, Gaithersburg, MD, USA) supplemented with 10% FBS (Gibco) and antibiotics (100 U/mL of penicillin and 100 μg/mL of streptomycin) at 37°C in a humidified atmosphere of 5% CO2. PEDV-CV777 (GenBank accession no. KT323979) stocked in our laboratory was propagated in Vero E6 cells as previously described (Hofmann and Wyler, 1988; Sun et al., 2015). To evaluate the anti-PEDV activity of porcine IFN-λ (Prosit Sole Biotechnology, Co., Ltd., Beijing, China), Vero E6 cells were pretreated with designated concentrations of IFN-λ for 12 h and then infected with PEDV (MOI of 0.1).

### Synthetic miRNAs, shRNAs, and Transfection

All of the miRNA mimics, miRNA inhibitors, and short hairpin RNAs (shRNAs) were synthesized by Gene Pharma (Shanghai, China). The miRNAs and shRNAs sequences are listed in Table 1. Lipofectamine 2000 (Invitrogen, Carlsbad, CA, USA) or Lipofectamine RNAiMAX (Invitrogen) was used to transfect cells with plasmid DNA or synthetic oligonucleotides according to the manufacturer's instructions. The cells were infected with PEDV as previously described after transfection for 24 h. The cells were harvested for quantitative real-time PCR (RT-qPCR) or treated with NP-40 lysis buffer for Western blotting after infection for 36 h.

**Table 1:** Sequences of miRNA mimics, inhibitors, and shRNAs.

**Small RNA**	**Sequence(5′-3′)**
miR-30c-5p	UGUAAACAUCCUACACUCUCAGC
miR-30c-5p inhibitor	GCUGAGAGUGUAGGAUGUUUACA
shSOCS1 #1	GTATGACAAGAGCCTCAAG
shSOCS1 #2	GTTCTCCGAACGTGTCACGT
shSOCS1 #3	GCGAGAGCTTCGACTGCCTCT

### Total RNA Isolation, Reverse Transcription, and qPCR

Total cellular RNA was isolated using an RNeasy Mini kit (Qiagen Sciences, Hilden, Germany) according to the manufacturer's instructions. Total RNA was extracted and reverse transcribed as previously described (Ma et al., 2018). For miRNA reverse transcription, cDNA was prepared with a miRNA First Strand cDNA Synthesis kit (Sangon Biotech, Shanghai, China). qPCR was conducted in triplicate with Power SYBR Green PCR Master Mix reagents (Takara) on a LightCycler480 II system (Thermo Fisher Scientific, Waltham, MA, USA) as previously described (Ma et al., 2018). The miRNA expression levels were normalized to the internal control of U6. The sequences of RT-qPCR primers for PEDV, IFN-λ, SOCS1, IFIT1, ISG15, MxA, GAPDH, miR-30c, and Uni-miR transcription are listed in Table 2. The results are presented as the means ± SEM from three separate trials.

**Table 2:** Sequences of primers used in the present study.

**Primer**	**Sequences(5′-3′)**
miR-30c-5p-qPCR-F	TGTAAACATCCTACACTCTCAGC
Uni-miR-qPCR-R	GCGAGCACAGAATTAATACGACTCAC
ISG15-qPCR-F	ACGCAGACTGTGGCCCACCT
ISG15-qPCR-R	CATTTATTTCCAGCCCTTGA
SOCS1-qPCR-F	CGCCCTCAGTGTGAAGATGG
SOCS1-qPCR-R	GCTCGAAGAGGCAGTCGAAG
GAPDH-qPCR-F	ATGGGGAAGGTGAAGGTCGG
GAPDH-qPCR-R	TCCTGGAAGATGGTGATGGG
IFN-λ-qPCR-F	ACCGCAGGAGTTGGCAAG
IFN-λ-qPCR-R	CCGGGGAAGACAGGAGAG
MxA-qPCR-F	CTGCTGCATCCCAACCTCTAT
MxA-qPCR-R	GGCGCACCTTCTCCTCGTACT
IFIT1-qPCR-F IFIT1-qPCR-R	GGTCTTGGAGGAGATTGA ATACAGCCAGGCATAGTT
SOCS1-EcoR I–F	CGGAATTCATGGTAGCACACAACCAGGTG
SOCS1-Kpn I–R	GGGGTACCTCATATCTGGAAGGGGAAGGAG
SOCS1-3′ UTR-Nhe I-F	CTAGCTAGCATTATTTCCTTGGAACCATGTG
SOCS1-3′ UTR-Xba I-R	GCTCTAGACACAGCAGAAAAATAAAGCCAG
SOCS1-3′ UTR-MT–F	CTTCATAGGGTCATATACCCAGTATCTTTGCACAAAC
SOCS1-3′ UTR-MT–R	TATATGACCCTATGAAGAGGTAGGAGGTACTGAGTTC

### miRNA Target Projections and Plasmid Construction

TargetScan Release 7.1 (http://www.targetscan.org) was used to predict the targets of miR-30c-5p. SOCS1 3′ UTRs as a prospective target was cloned and constructed as previously described (Ma et al., 2018). To construct the monkey SOCS1 expression vector, the full-length CDS region of monkey SOCS1 was amplified from Vero E6 cellular mRNA PCR and cloned into pCAGGS-HA vector (Clontech, Mountain View, CA, USA) using EcoR I and Kpn I restriction sites. The 3′UTR of SOCS1 (GenBank:100307052) was amplified and inserted into the pmirGLO luciferase reporter vector. The SOCS1 3′UTR mutant vector was produced by mutating five seed nucleotides using a site-directed mutagenic kit (Stratagene, La Jolla, CA, USA) according to the manufacturer's instructions. The constructed plasmids were verified by sequencing.

### Dual-Luciferase Assays

The luciferase activities were tested using a Dual-Luciferase Reporter Assay System (Promega, Madison, WI, USA) based on the manufacturer's instructions. Wild- or mutant-type SOCS1 3′UTR luciferase reporter vectors were co-transfected with miR-30c-5p mimics (miR-30c), mimic NC (NC), miR-30c-5p inhibitor (miR-30c-i), or inhibitor NC (NC-i) into Vero E6 cells for 24 h. Then pRL-TK was co-transfected with either miR-30c, NC, miR-30c-i, or NC-i for 24 h. The cells were collected and the luciferase activity was evaluated with a dual-luciferase reporter assay system (Promega). The pRL-TK vector expressing the Renilla luciferase gene was used as a normalization control.

### Immunofluorescence Assay (IFA)

Vero E6 cells were fixed with 4% paraformaldehyde for 30 min at 4°C and permeabilized with 0.2% Triton X-100 for 15 min, then blocked with blocking buffer (PBS with 5% FBS) for 2 h at 37°C. The cells were incubated with an anti-HA monoclonal antibody (Sigma-Aldrich, Munich, Germany, 1:5000) at 37°C for 2 h, followed by labeling with an Alexa Fluor 546 goat anti-mouse IgG antibody (Thermo Fisher Scientific, 1:500) at 37°C for 1 h. 4′,6-diamidino-2-phenylindole (DAPI, 1:100) was used to stain the cellular nuclei. The stained cells were visualized using an AMG EVOS F1 fluorescence microscope.

### Western Blotting

Vero E6 cells were lysed with NP-40 lysis buffer (Beyotime, China) supplemented with 0.1 mM of phenylmethylsulfonyl fluoride (PMSF) (Roche, Indianapolis, IN, USA). Target proteins were separated on SDS-PAGE gels then transferred onto nitrocellulose membranes (GE Healthcare, Chicago, IL, USA). After blocking with TBS-T containing 5% non-fat milk at room temperature (RT), the membranes were incubated with primary antibody at 4°C for 18 h. Antibodies included: β-actin (Sigma-Aldrich, 1:5000) and SOCS1 (Sigma-Aldrich, 1:500). The membranes were incubated with secondary antibody goat anti-mouse-HRP or goat anti-rabbit-HRP, diluted at 1:2000 for 1 h at room temperature, and visualized using an ECL system (Thermo Fisher). The results were analyzed using ImageJ software.

### Statistical Analysis

All of the data are described as the means ± the standard error of the mean (SEM). GraphPad Prism (GraphPad Software, Inc.) was used to analyze the data using Student's *t*-test. Each experiment was repeated three times. *P*-values <0.05 were considered significant: ^*^P < 0.05; ^**^P < 0.01; ^***^P < 0.001; ^****^P < 0.0001, and NS, not significant.

## Results

### PEDV Replicated Well Despite the Induction of Endogenous IFN-λ Responses During Late Infection

**Figure 1:**
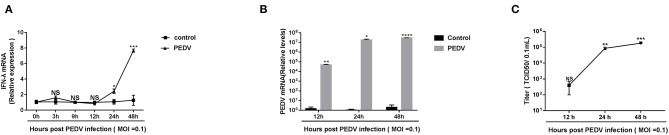
PEDV replicated successfully despite the induction of endogenous IFN-λ responses during late infection. **(A)** PEDV infection induced IFN-λ expression. IFN-λ expression was monitored in Vero E6 cells after infection with PEDV at MOIs of 0.1 and the results were normalized by the mock uninfected controls. **(B,C)** Kinetic curve of PEDV replication in Vero E6 cells. Vero E6 cells were inoculated with PEDV at an MOI of 0.1, and the level of PEDV infection compared to mock controls at 12, 24, and 48 h was quantified by RT-qPCR and TCID_50_. The results were obtained from three independent experiments. Mean ± SEM, *P < 0.05; **P < 0.01; ***P < 0.001; ****P < 0.0001, and NS, not significant.

Previous research showed that the pretreatment of porcine IFN-λ inhibits PEDV infection in Vero E6 cells and IPEC-J2 (Li et al., 2017). It is well-established that PEDV replicates efficiently in Vero E6 cells. To determine whether PEDV elicits an endogenous IFN-λ response in Vero E6 cells following infection, we initially infected Vero E6 cells with PEDV at MOI = 0.1 and monitored the IFN-λ expression. Compared with a mock uninfected control, PEDV did not increase the expression of IFN-λ transcripts as observed until 12 hpi, and then gradually induced IFN-λ expression, indicating that PEDV infection elicits type III IFN expression at the late stage of infection instead of the early stage of infection in the Vero E6 cells (Figure 1A), which was consistent with the results in porcine enteroids (Li et al., 2019). And PEDV propagated efficiently in Vero E6 cells by quantifying viral genomes and titers (Figures 1B,C). The virus titer increased up to 10^5^/0.1 mL at 48 hpi (Figure 1C). Interestingly, despite the increased expression of endogenous IFN-λ at the late stage of infection, the PEDV virus titer did not decrease. This indicates that there are mechanisms explored by PEDV to antagonize the endogenous IFN-λ ISG response at the late-stage infection.

### PEDV Infection Increased the Expression of SOCS1 in Vero E6 Cells

**Figure 2:**
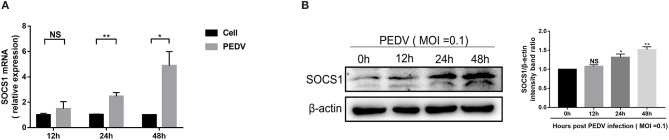
PEDV infection increased the expression of SOCS1 in Vero E6 cells. **(A,B)** PEDV infection increased the mRNA levels and protein levels of SOCS1. Vero E6 cells were infected with PEDV at MOI 0.1. The level of SOCS1 RNA was measured by RT-qPCR **(A)**. Cell lysates of Vero E6 cells were analyzed by Western blotting using an antibody against SOCS1 (left panel); β-actin was used as a loading control. The SOCS1 bands were normalized to β-actin using ImageJ software (right panel) **(B)**. The results were represented as Mean ± SEM of three independent experiments. *P < 0.05, **P < 0.01, and NS, not significant.

SOCS1, a typical member of the SOCS family of proteins, is a well-known negative feedback inhibitor of JAK/STAT signaling pathway induced by cytokines (Ma et al., 2018). To explore the underlying mechanisms exploited by PEDV to escape IFN-λ-induced antiviral responses, we initially assessed whether PEDV infection induces SOCS1 expression in Vero E6 cells. The mRNA levels of SOCS1 significantly increased following PEDV infection and displayed a time-dependent response (Figure 2A). The induction of SOCS1 by PEDV infection was further verified by SOCS1 Western blotting (Figure 2B).

### SOCS1 Counteracted the Anti-PEDV Activity of IFN-λ

**Figure 3:**
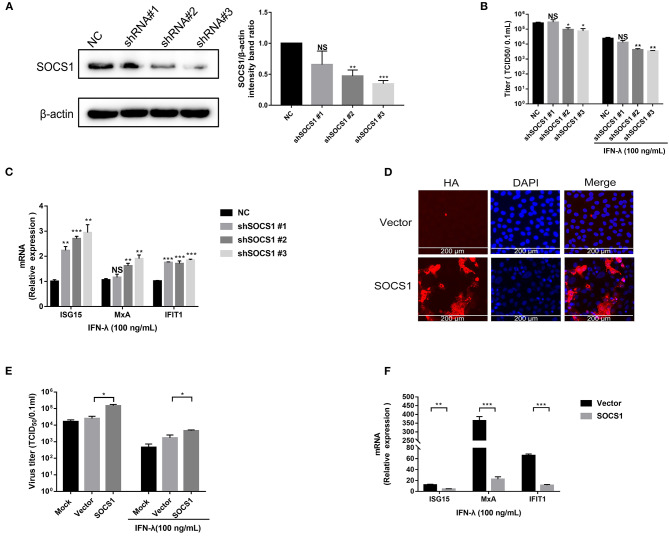
SOCS1 counteracted the anti-PEDV activity of IFN-λ. **(A)** The knockdown efficiency of shSOCS1 was determined by Western blotting (left panel); β-actin was used as a loading control. The SOCS1 bands were quantified using ImageJ software as normalized to β-actin (right panel). **(B)** Knockdown of SOCS1 decreased PEDV replication. Vero E6 cells were treated with 100 ng/mL of IFN-λ and transfected with NC or shSOCS1s, followed by infection with PEDV (MOI = 0.1). After 36 h, cell culture supernatants were harvested for virus titration. **(C)** Silencing of SOCS1 increased IFN-λ signaling induced by IFN-λ stimulation. Vero E6 cells were stimulated with IFN-λ 12 h after transfection with shSOCS1 #1, shSOCS1 #2, shSOCS1 #3, or scrambled control shRNA, and then the cells were collected for RT-qPCR analysis of ISG15, IFIT1, or MxA expression relative to that of GAPDH after 24 h of stimulation. **(D)** Transient expression of SOCS1 in Vero E6 cells. SOCS1 was cloned and expressed in the eukaryotic expression vector pCAGGS-HA. The overexpression efficiency of SOCS1 in Vero E6 cells was confirmed by IFA. **(E)** Transient expression of SOCS1 enhanced PEDV infection in Vero E6 cells. Vero E6 cells were stimulated with IFN-λ 12 h after the transient expression of SOCS1 promoted PEDV infection. Vero E6 cells were transfected with SOCS1-HA for 24 h and then infected with PEDV (MOI = 0.1). PEDV infection was determined by measuring PEDV titers. **(F)** Transient expression of SOCS1 disrupted the IFN-λ antiviral response. Vero E6 cells were stimulated with IFN-λ after being transfected with SOCS1 for 24 h. The cells were collected for RT-qPCR analysis of ISG15, MxA, and IFIT1 expression relative to that of GAPDH after 24 h of stimulation. Error bars, mean ±SEM. (*n* = 3 independent experiments). *P < 0.05, **P < 0.01, ***P < 0.001, and NS, not significant.

SOCS1 is a potent inhibitor of the type I and type II IFN signaling pathway (Skjesol et al., 2014). We next investigated whether SOCS1 suppresses IFN-λ-mediated antiviral activity. First, we silenced endogenous SOCS1 expression by specific shRNAs. SOCS1 shRNAs or a non-targeting shRNA (NC) were transfected into Vero E6 cells. The efficiency of SOCS1 knockdown was confirmed by Western blotting (Figure 3A). shSOCS1 #2 and #3 led to a 55 and 65% decrease in SOCS1 expression, respectively, compared with NC (Figure 3A). Silencing of endogenous SOCS1 by shSOCS1 #2 or #3 significantly reduced PEDV replication in Vero E6 cells without the addition of exogenous IFN-λ (Figure 3B). The decreased levels of PEDV infection were in line with the knockdown efficiency of SOCS1 shRNAs, indicating the specific effect of SOCS1 shRNAs. As previously reported, exogenous IFN-λ significantly inhibited PEDV infection, whereas silencing of endogenous SOCS1 by shSOCS1 #2 or #3 further enhanced the PEDV inhibition by IFN-λ in Vero E6 cells compared with untreated IFN-λ mock control (Figure 3B). The knockdown of endogenous SOCS1 by shSOCS1 #3 resulted in a more than 3.4-fold decrease in PEDV titer (Figure 3B) and degraded to 7.2-fold of PEDV titers with IFN-λ treatment in Vero E6 cells (Figure 3B), indicating that SOCS1 knockdown increases the antiviral effects of IFN-λ. Inconsistent with the viral results, SOCS1 knockdown increased the mRNA levels of ISG15, MxA, and IFIT1 (Figure 3C). We subsequently investigated the role of SOCS1 overexpression on the anti-PEDV effects of IFN-λ. The transient overexpression of SOCS1 in Vero E6 cells was verified by HA IFA (Figure 3D). As expected, SOCS1 overexpression substantially elevated PEDV infection (Figure 3E) and blunted the expression of ISG15, MxA, and IFIT1 (Figure 3F). Thus, these data indicate that SOCS1 counteracts the anti-PEDV activity of IFN-λ.

### PEDV Upregulated SOCS1 Expression by Modulating miR-30c-5p

**Figure 4:**
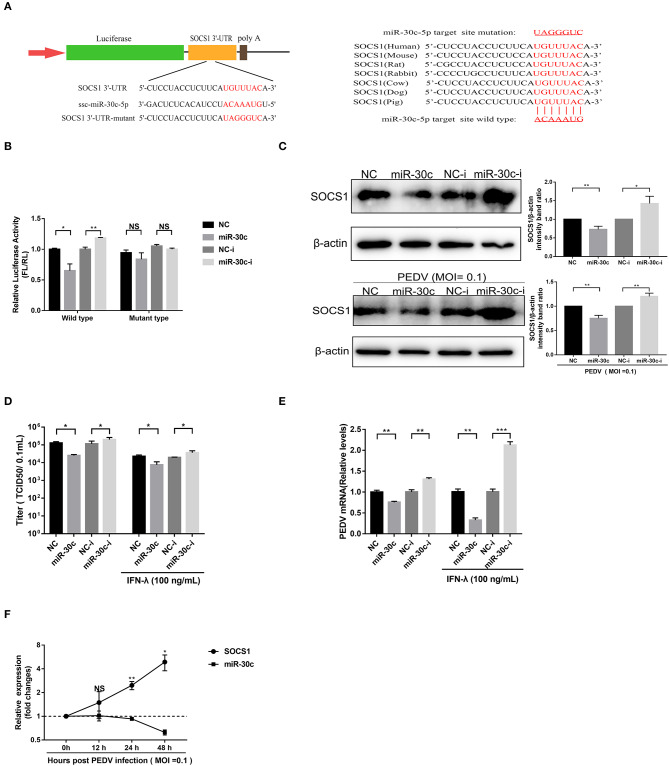
MiR-30c-5p targeted the 3′ UTRs of SOCS1. **(A)** Schematic diagram of the left panel is the predicted target sites of miR-30c-5p in the SOCS1 3′ UTRs of seven representative mammals. The predicted target sites and mutated target sites of miR-30c-5p are underlined and mutated as indicated (right panel). **(B)** Results of the luciferase assay. Vero E6 cells were co-transfected with SOCS1 wild-type or mutant luciferase vectors (500 ng) and 160 nM of miR-30c-5p mimics or NC mimics, miR-30c-5p inhibitor, or NC inhibitor, and the luciferase activity was analyzed at 24 h after transfection. FL, firefly luciferase; RL, Renilla luciferase. **(C)** The suppression of SOCS1 protein levels by miR-30c-5p under PEDV-uninfected and -infected conditions. Vero E6 cells were transfected as described in the legend for panel B for 24 h, followed by infection with PEDV (MOI=0.1) or mock infection with DMEM, and the samples were collected at 36 h for Western blotting of SOCS1 or β-actin. Quantifications were normalized to those of uninfected NC. **(D,E)** MiR-30c-5p increased the anti-PEDV activity of IFN-λ. After transfection with miR-30c-5p mimics or inhibitor for 24 h, cells were pretreated with IFN-λ or DMEM for 12 h and then infected with PEDV (MOI = 0.1) and harvested at 36 hpi for viral RNA quantification and TCID_50_. **(F)** The SOCS1 expression levels in Vero E6 cells were measured by RT-qPCR at 36 hpi at different MOIs. P values represent the difference from the mock-infected control for time kinetics, the SOCS1, and miR-30c-5p levels. Error bars, mean ± SEM. (*n* = 3 independent experiments). *P < 0.05, **P < 0.01, ***P < 0.001, and NS, not significant.

The porcine miR-30 family (five members: miR30a-e) has been demonstrated to modulate host type I IFN response during virus infection (Zhu et al., 2014; Zhang et al., 2016; Liu et al., 2018; Ma et al., 2018). The TargetScan (http://www.targetscan.org) prediction program indicated that SOCS1 was targeted by miR-30c-5p through a site in the 3′UTR conserved in the SOCS1 of seven representative mammals (Figure 4A). To investigate whether miR-30c-5p is involved in modulating IFN-λ signaling by directly targeting SOCS1 and downregulating endogenous SOCS1 expression, we conducted a computational analysis using TargetScan Release 7.1 (http://www.targetscan.org). The result showed that miR-30c could directly target the site on the 3′UTRs of SOCS1 (Figure 4A). We cloned the predicted target sites in porcine SOCS1 3′UTR, and constructed the firefly luciferase reporter vector of porcine SOCS1 3′UTR (Figure 4A). Overexpression of miR-30c-5p, the luciferase reporter containing the SOCS1 wild-type target sequence, decreased to ~65% relative to NC mimics, whereas the blockage of miR-30c by miR-30c inhibitor increased SOCS1 3′UTR luciferase activity. However, the mutation of the SOCS1 target 3′UTR site of miR-30c-5p disrupted the effects of miR-30c-5p on modifying the luciferase activity in Vero E6 cells relative to the NCs (Figure 4B). These results confirmed that miR-30c-5p directly targets the 3′ UTR of SOCS1. Consistent with the luciferase results, miR-30c-5p overexpression reduced SOCS1 expression measured by Western blotting (Figure 4C). Conversely, blockage of endogenous miR-30c-5p increased the expression of SOCS1 in Vero E6 cells compared with the NC inhibitor. To further validate the modulation of SOCS1 expression by miR-30c-5p during PEDV infection, we examined the expression of SOCS1 in PEDV-infected Vero E6 cells with overexpression or inhibition of miR-30c-5p, and found that the expression pattern of SOCS1 in PEDV infected Vero E6 cells was similar to that in PEDV-uninfected E6 cells (Figure 4C). Taken together, these data demonstrated that miR-30c-5p downregulates the expression of SOCS1 by directly targeting SOCS1 3′UTR.

To verify whether PEDV escape the IFN-λ antiviral signaling through miR-30c-5p mediated modification of SOCS1 expression, we then explored the effect of miR-30c-5p on PEDV infection and IFN-λ antiviral signaling. Transient miR-30c expression reduced PEDV titers and promoted IFN-λ anti-PEDV activity compared with the mock control NCs, whereas miR-30c-5p inhibitor significantly increased PEDV infection and undermined the anti-PEDV activity of IFN-λ (Figures 4D,E). Furthermore, the SOCS1 expression increased starting at 12 hpi and substantially increased at 24 hpi, which was inversely correlated with the kinetic expression profiles of miR-30c-5p (Figure 4F). In agreement with the kinetics pattern of miR-30c-5p and SOCS1 in Vero E6 cells, PEDV infection reduced the levels of miR-30c-5p and increased SOCS1 expression in IPEC-J2 starting at 12 h post-infection (data not shown). Collectively, PEDV infection upregulates SOCS1 expression by modulating host miR-30c-5p abundance at the late stage of infection.

### miR-30c-5p Facilitated PEDV Infection via Antagonizing IFN-λ Signaling by Targeting SOCS1

**Figure 5:**
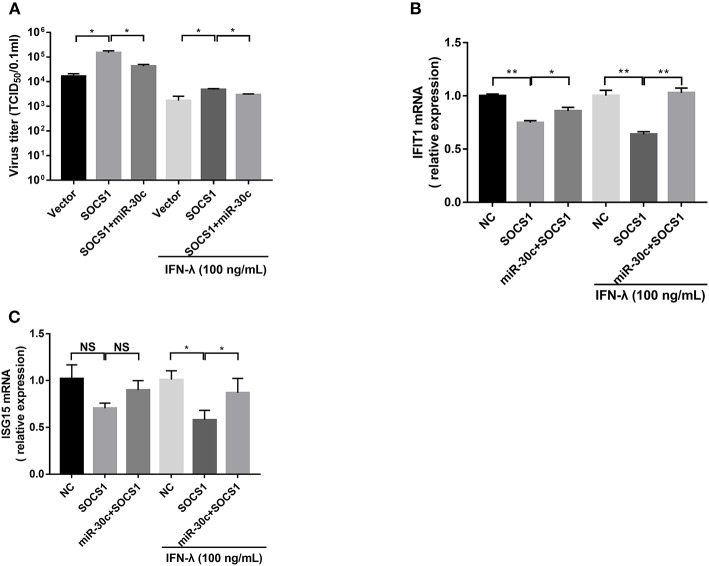
Mir-30c-5p inhibited the infection of PEDV by regulating the expression of SOCS1. **(A)** SOCS1 overexpression increased PEDV infection and undermined the anti-PEDV activity of IFN-λ. Vero E6 cells were transfected as described with pCAGGS-HA, pCAGGS-SOCS1, and miR-30c-5p for 24 h, followed by incubation with porcine IFN-λ (100 ng/ml) or DMEM for 12 h. The cells then were infected with PEDV at an MOI of 0.1; PEDV infection was determined at 36 hpi. **(B,C)** miR-30c-5p abolished the impairment of the overexpression of SOCS1 to IFN-λ signaling under IFN-λ-stimulated or PEDV-infected conditions. E6 cells were treated as described in the legend for panel A. The cells were collected for RT-qPCR analysis of IFIT1 and ISG15 expression relative to that of GAPDH. Error bars, mean ± SEM (*n* = 3 independent experiments). *P < 0.05, **P < 0.01, and NS, not significant.

To further verify whether PEDV escape IFN-λ response through the miR-30c-5p/SOCs1 axis, we co-transfected miR-30c-5p with SOCS1 and measured the replication of PEDV with or without IFN-λ treatment. SOCS1 overexpression promoted PEDV replication with or without IFN-λ treatment. MiR-30c-5p largely abolished the role of SOCS1 in promoting PEDV replication, and the effect was more pronounced in the presence of IFN-λ stimulation (Figure 5A). Consistent with this, SOCS1 inhibited IFN downstream ISGs expression such as IFIT1 and ISG15 expression (Figures 5B,C), whereas overexpression of miR-30c-5p abrogated the ISG inhibition of SOCS1, which was more pronounced in the presence of IFN-λ priming (Figures 5B,C). In summary, these data indicate that PEDV escapes the response of IFN-λ through the miR-30a-5p/SOCS1 axis.

## Discussion

IFN-λ is an antiviral innate cytokine induced by virus infection that plays vital roles in controlling mucosal infection (Blumer et al., 2017). We and other groups previously showed that IFN-λ substantially inhibits PEDV (Li et al., 2017; Zhang et al., 2018). However, whether PEDV has evolved a mechanism to counteract endogenous IFN-λ just as PEDV does the type I IFN response remains unclear. In this study, we found that PEDV propagated well despite the significant production of endogenous IFN-λ induced at the late stage of infection in Vero E6 cells, indicating that PEDV escaped the IFN-λ response at the late stage of infection not through suppressing IFN-λ production. We further defined the mechanism that PEDV counteracted IFN-λ-elicited antiviral ISG responses by exploiting the miR-30c-5p/SOCS1 axis.

PEDV has evolved multiple strategies to escape type I IFN response. Whether PEDV exploits similar mechanisms to counteract type III IFN remains elusive. One previous study demonstrated that PEDV escaped type III IFN by suppressing IRF1-mediated IFN-λ production through PEDV viral nsp1 protein (Zhang et al., 2018). In that study, PEDV actually upregulates IFN-λ expression at 3 h post-infection and then decreased to minimal levels of IFN-λ expression until 12 hpi (Zhang et al., 2018). In agreement with this, we did not observe increased IFN-λ expression at 12 hpi (Figure 1A). They did not show the IFN-λ expression at the late stage of PEDV infection. In the current study, we observed that PEDV elicited substantially increased IFN-λ expression in Vero E6 cells only after 24 hpi (Figure 1A), which is consistent with the results observed in porcine enteroids following PEDV infection (Li et al., 2019), indicating that PEDV has evolved mechanisms to escape IFN-λ antiviral response instead of IFN-λ production at the late stage of infection. It is possible that PEDV exploits varying strategies at different infection stages. This is also observed in other RNA viruses such as influenza virus (Chung et al., 2018). To prevent over-activation of the IFN signaling pathways, the host evolves a few negative regulators of IFN signaling, and SOCS1 is one of the canonical inhibitors of IFN signaling (Shao et al., 2013). SOCS1 has been reported to be exploited by multiple viruses to abrogate IFN antiviral signaling (Shao et al., 2013; Wei et al., 2014; Ma et al., 2018). We showed that PEDV significantly induced the expression of SOCS1 at the late stage of infection (Figure 2). As expected, increased SOCS1 impaired the antiviral ISGs expression and impaired the anti-PEDV activity of IFN-λ (Figure 3). This is in agreement with the results of TGEV, another swine alphacoronavirus (Ma et al., 2018). Therefore, unlike previously published studies with the modification of IFN production mediated by viral proteins such as nsp1, our study found that PEDV largely evades innate immunity of IFN-λ by modulating the antiviral signal of IFN-λ rather than manipulating the production of IFN-λ at the late stage of infection.

MiRNA plays a vital role in regulating gene expression through post-transcription modification. Increasing evidence demonstrates that viruses escape IFN antiviral activity for optimal infection by modifying the cellular abundance of miRNA targeting vital components of the IFN response (Zhu et al., 2014; Zhang et al., 2016; Liu et al., 2018; Ma et al., 2018). JEV infection downregulates the expression of miRNA miR-432, which directly targets the suppressor of cytokine signaling protein 5 (SOCS5) and manipulates the JAK-STAT1 signaling cascade (Sharma et al., 2016). The miR-30 family has been reported to target SOCS family members and manipulate the JAK/STAT signaling pathway (Zou et al., 2017; Ma et al., 2018; Yuan et al., 2019). In this study, we showed that PEDV infection suppressed miR-30c-5p expression, which was conversely related to SOCS1 expression during PEDV infection (Figure 4). Just as other members of miR-30, miR-30c-5p specifically targeted the 3′ UTR of SOCS1 and inhibited SOCS1 expression (Kobayashi et al., 2012; Ma et al., 2018; Yuan et al., 2019) (Figure 4). However, the mechanism of PEDV decreasing miR-30a-5p remains unclear and deserves further study.

In summary, we determined that PEDV escaped IFN-λ response at the late stage of infection by downregulating miR-30c-5p, thus increasing SOCS1 expression. Therefore, unlike previously published studies with defined mechanisms such as nsp1, we demonstrated that PEDV escapes IFN-λ response through another pathway of the miR-30c-5p/SOCS1 axis. Our results highlight the important role of miR-30c-5p in the regulation of interferon pathways during PEDV infection, improve the current knowledge of PEDV infection, and expand the role of micro-RNA in viral infection.

## Data Availability Statement

The datasets generated for this study are available on request to the corresponding author.

## Author Contributions

PL, CW, LS, and WX designed the research studies and analyzed and interpreted the data. CW, LS, SQ, MX, KW, FF, LW, ZW, and LF conducted the experiments and collected the data. PL, CW, and LS drafted the manuscript. All of the authors contributed revisions.

## Conflict of Interest

The authors declare that the research was conducted in the absence of any commercial or financial relationships that could be construed as a potential conflict of interest.
